# *Streptococcus oligofermentans* Inhibits *Streptococcus mutans* in Biofilms at Both Neutral pH and Cariogenic Conditions

**DOI:** 10.1371/journal.pone.0130962

**Published:** 2015-06-26

**Authors:** Xudong Bao, Johannes Jacob de Soet, Huichun Tong, Xuejun Gao, Libang He, Cor van Loveren, Dong Mei Deng

**Affiliations:** 1 Department of Cariology and Endodontology, Peking University School and Hospital of Stomatology, Beijing, China; 2 Department of Preventive Dentistry, Academic Centre for Dentistry Amsterdam, University of Amsterdam and VU University Amsterdam, Amsterdam, The Netherlands; 3 State Key Laboratory of Microbial Resources, Institute of Microbiology, Chinese Academy of Sciences, Beijing, China; 4 State Key Laboratory of Oral Diseases, West China Hospital of Stomatology, Sichuan University, Chengdu, China; LSU Health Sciences Center School of Dentistry, UNITED STATES

## Abstract

Homeostasis of oral microbiota can be maintained through microbial interactions. Previous studies showed that *Streptococcus oligofermentans*, a non-mutans streptococci frequently isolated from caries-free subjects, inhibited the cariogenic *Streptococcus mutans* by the production of hydrogen peroxide (HP). Since pH is a critical factor in caries formation, we aimed to study the influence of pH on the competition between *S*. *oligofermentans* and *S*. *mutans* in biofilms. To this end, *S*. *mutans* and *S*. *oligofermentans* were inoculated alone or mixed at 1:1 ratio in buffered biofilm medium in a 96-well active attachment model. The single- and dual-species biofilms were grown under either constantly neutral pH or pH-cycling conditions. The latter includes two cycles of 8 h neutral pH and 16 h pH 5.5, used to mimic cariogenic condition. The 48 h biofilms were analysed for the viable cell counts, lactate and HP production. The last two measurements were carried out after incubating the 48 h biofilms in buffers supplemented with 1% glucose (pH 7.0) for 4 h. The results showed that *S*. *oligofermentans* inhibited the growth of *S*. *mutans* in dual-species biofilms under both tested pH conditions. The lactic acid production of dual-species biofilms was significantly lower than that of single-species *S*. *mutans* biofilms. Moreover, dual-species and single-species *S*. *oligofermentans* biofilms grown under pH-cycling conditions (with a 16 h low pH period) produced a significantly higher amount of HP than those grown under constantly neutral pH. In conclusion, *S*. *oligofermentans* inhibited *S*. *mutans* in biofilms not only under neutral pH, but also under pH-cycling conditions, likely through HP production. *S*. *oligofermentans* may be a compelling probiotic candidate against caries.

## Introduction

The human oral cavity harbours a dynamic microbial community, which consists of more than 700 bacterial species [1]. In the healthy situation, this community maintains a healthy microbial homeostasis, through a dynamic balance of synergistic and antagonistic microbial interactions. Disturbance of this homeostasis can lead to shifts in microbial composition and eventually cause diseases *e*.*g*. dental caries [2]. Dental caries is chemical dissolution of the dental hard tissues by the acid produced when bacteria metabolise dietary carbohydrates. Under prolonged low-pH environment, the outgrowth of acidogenic and aciduric species such as mutans streptococci and lactobacilli shifts the microbial composition and enhances the dissolution of the dental hard tissue, eventually causing caries [3,4]. One of the key concepts in the prevention and treatment of dental caries is to prevent disturbance of or to re-establish a healthy microbial homeostasis [3]. Clinical studies have indicated that in the dental biofilms high numbers of non-mutans streptococci, such as *Streptococcus sanguinis* and *Streptococcus gordornii*, were often associated with low numbers of cariogenic bacteria *Streptococcus mutans* and this association was mostly observed in healthy subjects, while the inverse was typically found in subjects with caries [5,6]. These findings have emphasised the potential of caries prevention via modulating oral microbial ecology.

Among the commensal oral non-mutans streptococci, *Streptococcus oligofermentans* has several interesting traits. It was frequently isolated from caries-free subjects or healthy non-carious tooth surfaces [7,8]. It produced less acid from glucose than *S*. *mutans* [8] and inhibited the growth of *S*. *mutans* [9]. With molecular techniques, Tong *et al* [9] demonstrated that *S*. *oligofermentans* inhibited the growth of *S*. *mutans* through the production of hydrogen peroxide (HP) both in suspensions and in biofilms. *S*. *oligofermentans* employs three types of enzymes, pyruvate oxidase (POX), lactate oxidase (LOX) and L-amino acid oxidase, to produce HP [9,10,11]. The synergistic action of POX and LOX maximized the HP production of *S*. *oligofermentans* [10]. The ability of *S*. *oligofermentans* producing HP from lactic acid is particularly interesting, since lactic acid is the major organic acid produced by dental biofilms. This trait of *S*. *oligofermentans* may provide dual benefits: minimising pH drop by converting lactic acid into HP and inhibiting the cariogenic bacteria *S*. *mutans* through HP production. Therefore, *S*. *oligofermentans* may be a good probiotic candidate for maintaining healthy oral microflora.

Although several studies have reported that *S*. *oligofermentans* could inhibit the growth of *S*. *mutans* in a dual-species biofilm [8,10,11], some characteristics of the biofilm model used in these studies may limit the clinical relevance of their findings: firstly, the studied biofilms were “bottom-biofilms”. These biofilms mostly contain sedimented cells, which are not incorporated by active attachment, while active attachment is a prerequisite for oral biofilm formation. Secondly, the pH in the bottom-biofilm model was unknown and not controlled. Environmental factors, such as the presence of oxygen, sugar availability and pH, were shown to greatly affect the HP production of *S*. *oligofermentans*, thereby its inhibition on the growth of *S*. *mutans* [12,13]. A previous study demonstrated that the inhibitory effect of *S*. *oligofermentans* on *S*. *mutans* decreased with the decreasing pH value. At pH 5.5, no interaction between two species was observed in the agar competition assay [12]. Since *S*. *mutans* was known to be aciduric and to be able to outcompete other bacterial species at cariogenic condition (pH 5.5), the above findings seemed to suggest the limitations of *S*. *oligofermentans* in maintaining healthy microflora at cariogenic conditions. As only planktonic cultures were tested in the previous study, it is relevant to re-evaluate the influence of pH in a biofilm model that allows bacterial active attachment.

The aims of this study are to establish a pH-controllable active-attachment biofilm model and to explore the competition between *S*. *mutans* and *S*. *oligofermentans* in biofilms under two different pH conditions, constantly neutral pH and pH-cycling. The pH-cycling included a period of 8 h at neutral pH and a period of 16 h at pH 5.5, with the intention to mimic cariogenic conditions that dental biofilms often encounter.

## Materials and Methods

### Bacterial strains and growth conditions

The strains used in this study were *Streptococcus mutans* UA159 and *Streptococcus oligofermentans* LMG22279 [7]. Both bacterial strains were grown anaerobically (90% N_2_, 5% CO_2_, 5% H_2_) at 37°C. Biofilms were grown in a modified semi-defined biofilm medium (BM), which contains 10 mM (NH_4_)_2_SO_4_, 35 mM NaCl, 2 mM MgSO_4_·7H_2_O and was supplemented with filter-sterilised vitamins (0.04 mM nicotinic acid, 0.1 mM pyridoxine HCl, 0.01 mM pantothenic acid, 1 μM riboflavin, 0.3 μM thiamine HCl, and 0.05 μM D-biotin), amino acids (4 mM L-glutamic acid, 1 mM L-arginine HCl, 1.3 mM L-cysteine HCl, and 0.1 mM L-tryptophan), and 0.3% (wt/vol) yeast extract [14].

To prepare BM of pH 7.0, 76 mM K_2_HPO_4_ and 15 mM KH_2_PO_4_ were added to the medium. To prepare BM of pH 5.5, 30 mM MES buffer was added to the medium. For pre-cultures, BMG was prepared by adding 0.4% of glucose to BM and for biofilm growth, BMS was prepared by adding 0.2% of sucrose to BM. This sucrose concentration was chosen because it could promote biofilm formation without causing pH changes.

### Biofilm growth

**Fig 1:**
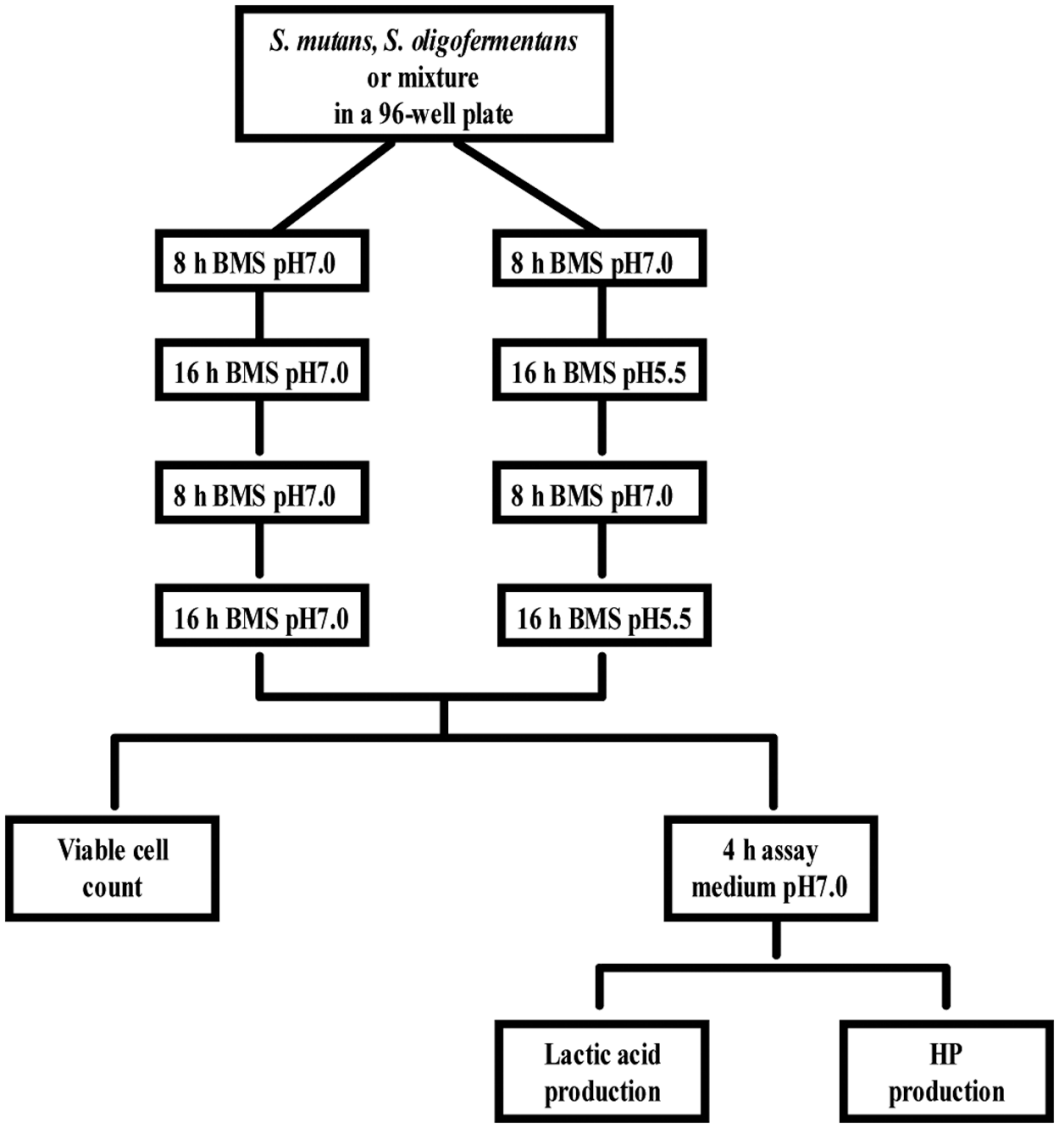
The schema of the biofilm experiment.

Biofilms were grown in an active attachment model [15]. This model consists of a standard 96-well microtiter plate and a lid with an identical number of polystyrene pegs that fit into wells (Nunc, Roskilde, Denmark). This model was chosen to examine exclusively the actively adhered biofilms, instead of bacterial sedimentation in the 96-well microtiter plate, and to retain the 96-well high-throughput advantage for testing multiple variables. Single or dual-species biofilms were grown for 48 h before further analysis. In detail, to grow single-species biofilms, overnight (16 h) *S*. *mutans* and *S*. *oligofermentans* cultures in BMG were diluted to a final OD_600nm_ of 0.04 in fresh BMS (pH 7.0) and 200 μl of each cell suspension was dispensed into a 96-well plate. To grow dual-species biofilms, the overnight cultures of each strain were diluted to a final OD_600nm_ of 0.08 in BMS (pH 7.0) and mixed at 1:1 ratio before dispensing 200 μl into the 96-well plate. This mixture contains 4.6 x 10^6^ (± 2.7 x 10^6^) CFU/ml of *S*. *mutans* and 1.7 x 10^7^ (± 7.4 x10^6^) CFU/ml of *S*. *oligofermentans*. The plate was then covered with the lid containing pegs and incubated for 8 h. Thereafter, the pegs were rinsed with sterile distilled water to remove non-adherent bacterial cells. After rinsing, half of the pegs were inserted in BMS of pH 7.0, while the other half was inserted in BMS of pH 5.5, and both were further incubated for 16 h. Subsequently all pegs were rinsed with sterile distilled water and inserted again in BMS of pH 7.0. After another 8 h incubation, the pegs were inserted in either BMS of pH 7.0 or BMS of pH 5.5 for another 16 h. The 48 h biofilms formed on the pegs were collected for viable cell counts and were examined for their capability to produce lactic acid and HP. The schema of biofilm growth is illustrated in Fig 1.

In each experiment, a total of 6 groups (single-/dual-species groups, grown under neutral pH or pH-cycling condition) were tested. Each group generally contained 8 biofilms replicates. Four replicates were used for viable cell counts and four were used for the measurement of lactic acid and HP production. The experiment was repeated 3 times.

### Viable cell counts

Each individual peg with biofilms was carefully cut off with a sterile scalpel without disturbing the biofilms and placed in 1ml CPW (5 g yeast extract, 1 g peptone, 8.5 g NaCl, 0.5 g L-cysteine hydrochloride per liter, adjusted to pH 7.3). Biofilms were dispersed by sonication on ice 60 times for 1 s at an amplitude of 40 W (Vibra cell, Sonics & Materials Inc., USA). Undiluted samples or serially diluted samples (100 μl) were plated onto Brain Heart Infusion (BHI) agar plates. The plates were incubated for 3 d. The colonies were counted and recorded as Colony Forming Unit (CFU). Since the morphologies of *S*. *mutans* and *S*. *oligofermentans* were distinct on BHI agar plates, the CFUs of *S*. *mutans* and *S*. *oligofermentans* in the dual-species biofilm samples were recorded separately based on the colony morphology. The images of the colonies are provided in S1 and S2 Figs. The detection limit of this viable cell count method is 100 CFU per sample.

### HP and lactic acid quantification

The capability of HP and lactic acid production of 48 h biofilms was examined by inserting the pegs with biofilms into a 96-well plate, filled with 200 μl per well buffered assay medium (pH 7.0) at 37°C for 4 h. The assay medium contained most of the components of BM except yeast extract, since no further biofilm growth was desired during the incubation. Glucose (1%) was added to the assay medium in order to trigger lactic acid production. After incubation, 50 μl assay medium was immediately used for HP measurement. The rest of the medium was stored at -20°C for lactic acid quantification.

HP was quantified by an enzymatic assay with modifications [16]. In detail, 50 μl of culture medium was added to 45 μl of solution containing 2.5 mM 4-aminoantipyrine (4-amino-2, 3-dimethyl-1-phenyl-3-pyrazolin-5-one; Sigma-Aldrich, St. Louis, MO, USA) and 0.17 M phenol in a 96-well plate. This reaction mixture was incubated for 5 min at room temperature; thereafter horseradish peroxidase (Sigma-Aldrich, St. Louis, MO, USA) was added at a concentration of 640 mU ml^-1^ in 0.2 M potassium phosphate buffer (pH 7.2). After 4 min, the absorbance was recorded at 510 nm in a spectrophotometer (Perkin Elmer, Norwalk, CT, USA). HP concentration of each sample was calculated from a standard curve generated with known concentrations of HP (Sigma-Aldrich, St. Louis, MO, USA).

Lactic acid was measured with an enzymatic-spectrophotometric method [17]. The principle of the method is based on the enzymatic conversion of L-lactate to pyruvate with concomitant conversion of NAD to NADH, the increase in absorbance at 340 nm being proportional to NADH formation.

### HP production of *S*. *oligofermentans* suspension cells at pH 7.0 or pH 5.5

To understand how environmental pH influenced the ability of HP production of *S*. *oligofermentans*, we chose to carry out the experiment on suspension cells since these cells were more homogenous and better controlled than the biofilm cells. Overnight *S*. *oligofermentans* culture grown in BMG (pH 7.0) was centrifuged and resuspended in either BM pH7.0 or BM pH5.5, without any addition of sucrose. The cell density was adjusted to an OD_600nm_ of 0.9. The resuspensions were incubated anaerobically for either 4 or 16 h. The ability of these suspension cells to produce HP was examined in the same way as the biofilms were tested. In detail, the resuspensions were centrifuged, incubated in the buffered assay medium for 4 h. The supernatants were used for HP quantification (the procedure is described above). The *S*. *oligofermentans* cells before and after 4 or 16 h of incubation were also subjected to viable cell counts. This experiment was repeated 3 times. Duplicate samples per group were included in each experiment.

### Statistical analysis

The data were analysed with the Statistical Package for Social Science (SPSS, Version 17.0, Chicago, IL, USA). One-way ANOVA was used to evaluate the differences in CFU, HP and lactic acid concentration between single- and dual-species biofilms. Specifically, independent-samples *t* test was used to examine the influence of pH (neutral pH vs. pH-cycling) on the variables. The CFU counts were log transformed before the statistical tests. *p<*0.05 was considered significant.

## Results

### Viable cell counts of single- and dual-species biofilms

Both *S*. *mutans* and *S*. *oligofermentans* were able to form biofilms alone and together in the presence of 0.2% sucrose. At every refreshment point the pH of the spent medium was monitored. After 16 h incubation, the pH of phosphate buffered biofilm medium (pH7.0) had remained constant, but the pH of MES buffered medium (pH 5.5) dropped 0.5 unit.

**Fig 2:**
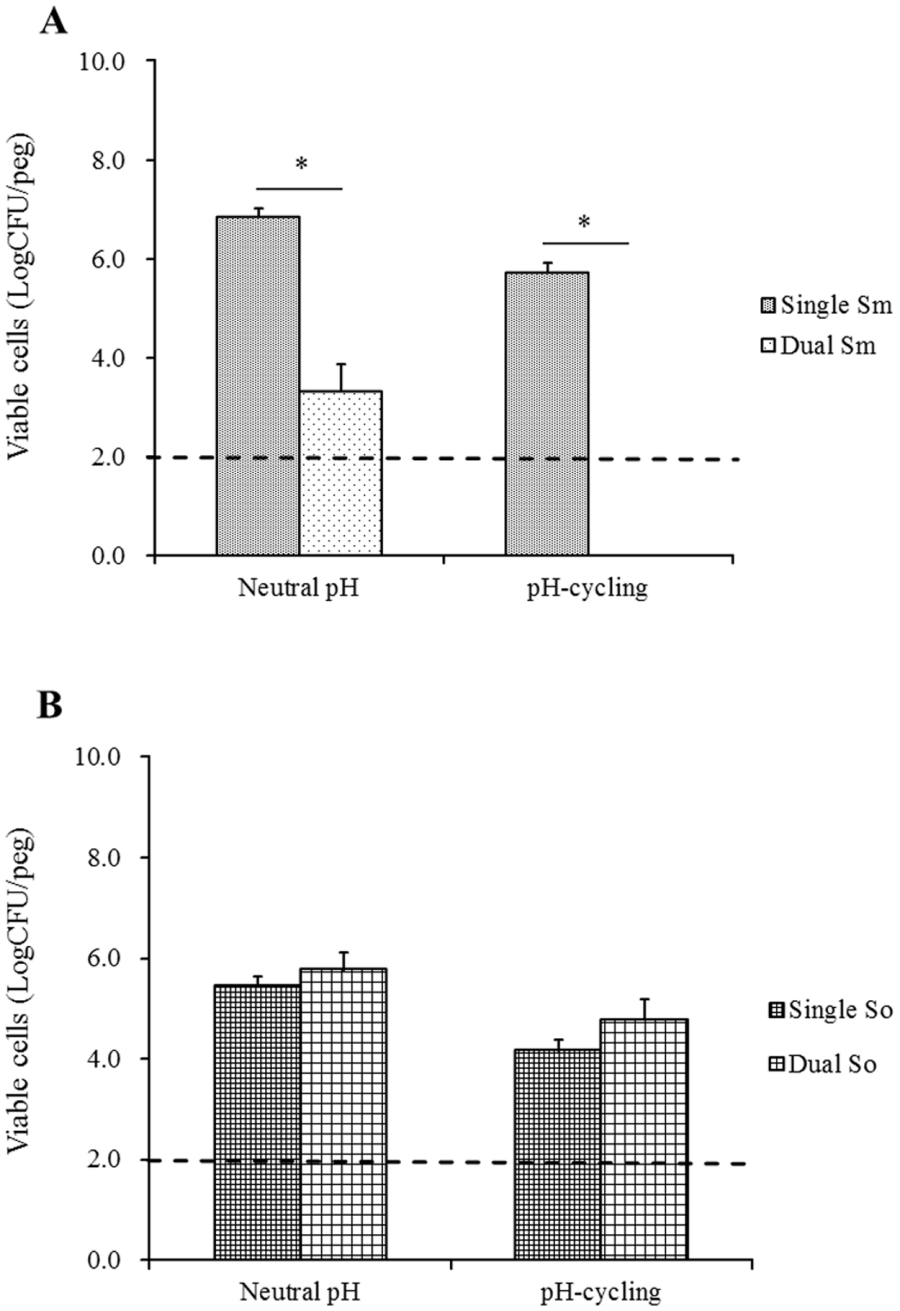
Viability of single-species and dual-species 48 h biofilms. Biofilms were grown under constantly neutral pH or pH-cycling (8 h pH 7.0 and 16 h pH 5.5) conditions. The viability of the biofilms was evaluated by CFU counts. **A.** Viable cell counts of *S*. *mutans* biofilms. Single Sm: *S*. *mutans* single-species biofilms; Dual Sm: *S*. *mutans* counts in dual-species biofilms. **B.** Viable cell counts of *S*. *oligofermentans* biofilms. Single So: *S*. *oligofermentans* single-species biofilms; Dual So: *S*. *oligofermentans* counts in dual-species biofilms. The dash line indicates the detection limit. Four replicates were used for viable cell counts in each experiment. The experiment was repeated 3 times. * indicates the significant difference between Single Sm and Dual Sm, *p* < 0.05.

Fig 2 displays the CFU counts of single- and dual-species biofilms. Compared to constantly neutral pH condition, pH 7.0–5.5 cycling condition generally lead to one log reduction in the total cell counts of 48 h biofilms. The presence of *S*. *oligofermentans* in dual-species biofilms significantly suppressed the growth of *S*. *mutans*. The cell counts of *S*. *mutans* dropped from 10^6^ CFU/peg in single-species biofilms to 10^3^ CFU/peg in dual-species biofilms at constantly neutral pH, and dropped from 10^5^ CFU/peg to under the detection limit (10^2^ CFU/peg) at pH-cycling conditions. However, the presence of *S*. *mutans* in dual-species biofilms did not seem to affect *S*. *oligofermentans* cell counts at tested pH conditions. The number of *S*. *oligofermentans* viable cells in dual-species biofilms was similar to that in single-species biofilms at either tested pH.

### Lactic acid concentration of single- and dual-species biofilms

**Fig 3:**
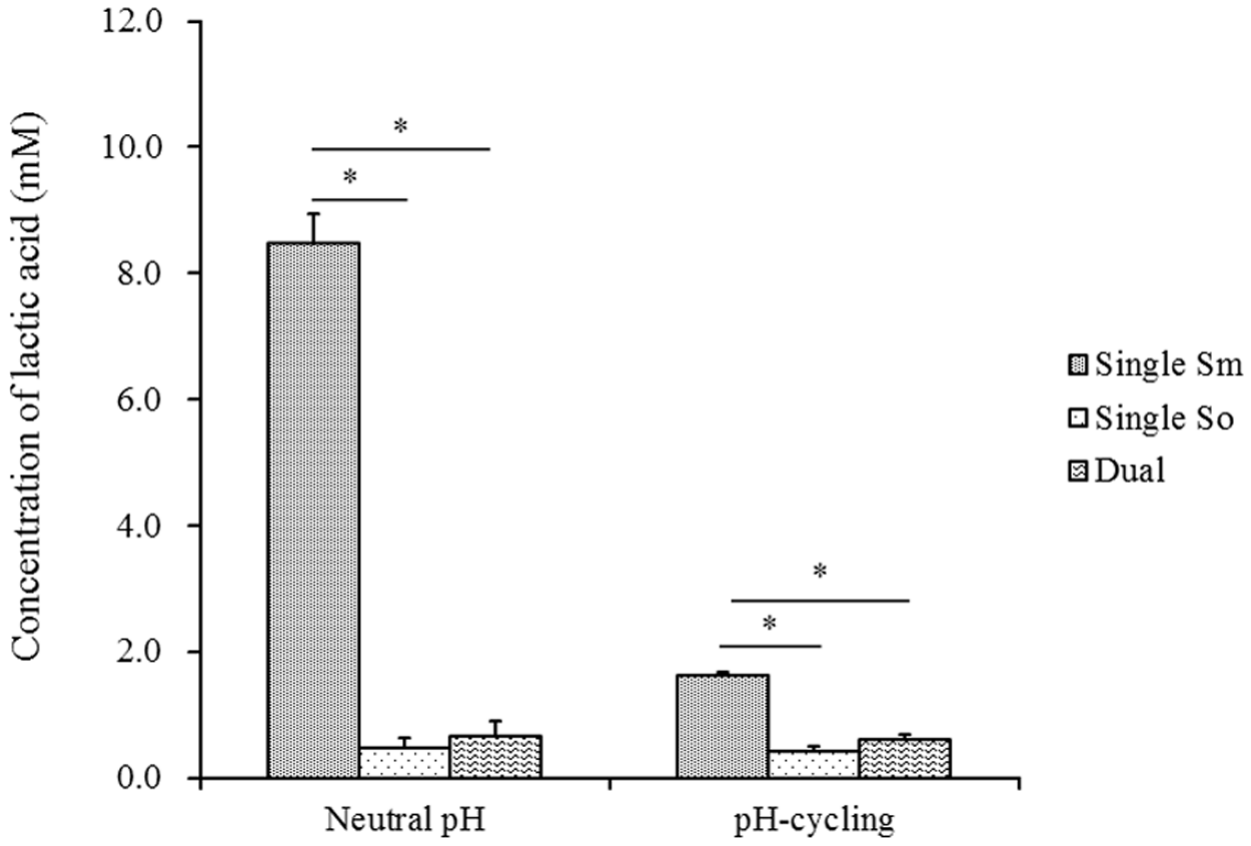
Lactic acid concentration of single-species and dual-species 48 h biofilms. Biofilms were grown under constantly neutral pH or pH-cycling conditions. Before quantification of lactic acid concentration, the biofilms were incubated in the assay medium (pH 7.0), supplemented with 1% glucose for 4 h. Single Sm: *S*. *mutans* single-species biofilms; Single So: *S*. *oligofermentans* single-species biofilms; Dual: dual-species biofilms. Four replicates were used for lactic acid quantification in each experiment. The experiment was repeated 3 times. * indicates the significant difference between Single Sm and the other two groups (Single So and Dual), *p* < 0.05.

The growth pH conditions affected only the lactic acid concentration of single-species *S*. *mutans* biofilms (Fig 3). The pH-cycling condition, compared to neutral pH condition, significantly reduced lactic acid production of single-species *S*. *mutans* biofilms (*p<*0.05). The lactic acid concentration in dual-species biofilms was significantly lower than that in single-species *S*. *mutans* biofilms. The reduction was around 12-fold under constantly neutral pH and 2.7-fold under pH-cycling condition. Similar level of acid concentration was observed in dual-species and single-species *S*. *oligofermentans* biofilms.

### HP production of single- and dual-species biofilms

**Fig 4:**
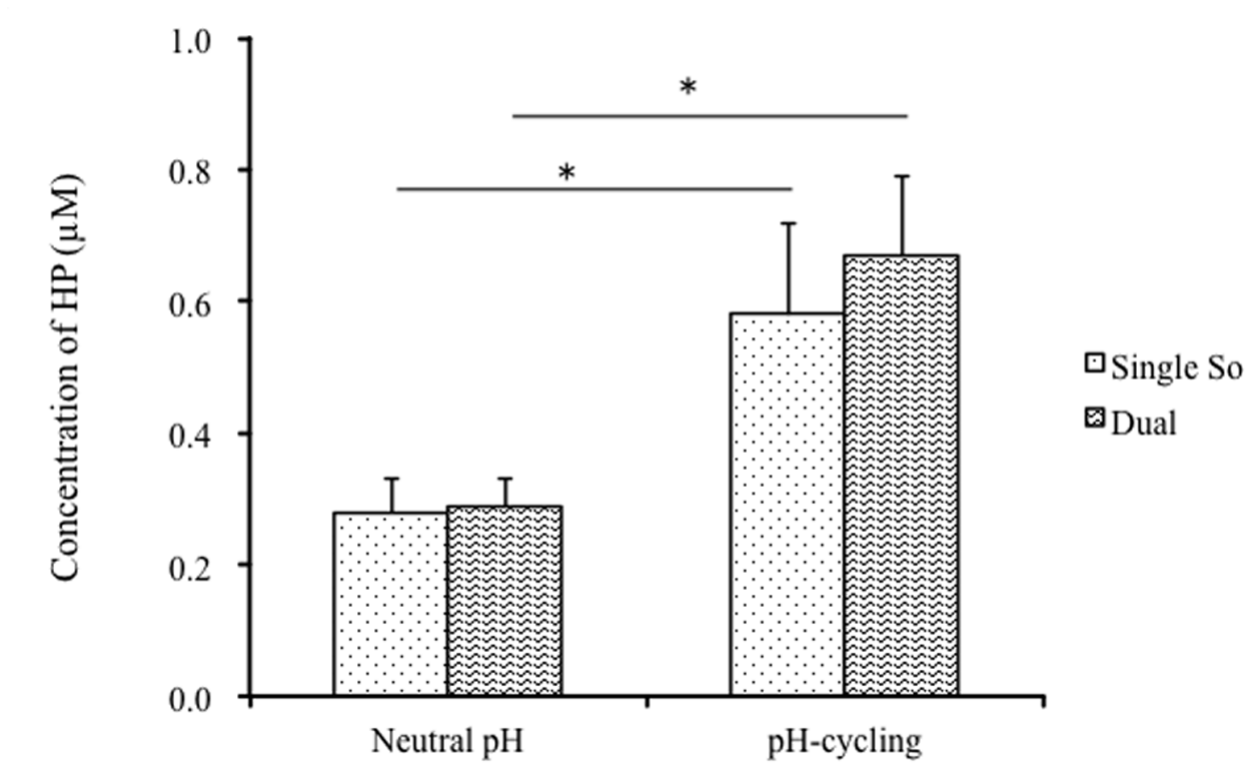
Hydrogen peroxide (HP) production of single-species and dual-species 48 h biofilms. Biofilms were grown under constantly neutral pH or pH-cycling conditions. The HP production was quantified after the biofilms were incubated in the assay medium (pH 7.0), supplemented with 1% glucose for 4 h. Single So: *S*. *oligofermentans* single-species biofilms; Dual: dual-species biofilms. Four replicates were used for HP production in each experiment. The experiment was repeated 3 times. * indicates the significant difference between constantly neutral pH and pH-cycling conditions, *p* < 0.05.

No HP production was measured for single-species *S*. *mutans* biofilms. We also examined the suspension cells of *S*. *mutans* UA159 grown in either BHI or BMS (pH 7.0) and found no HP production. This experiment was performed twice with duplicate samples (data not shown). Therefore HP in the biofilms was produced by *S*. *oligofermentans* alone (Fig 4). There were no differences in HP production between dual-species and single-species *S*. *oligofermentans* biofilms. The HP production in the biofilms grown at the pH-cycling condition was 2-fold higher than those in the biofilms grown at constantly neutral pH.

### HP production by *S*. *oligofermentans* suspension cells

To further explore if the increased HP production of *S*. *oligofermentans* under the pH-cycling condition was stress-related, we measured HP production of *S*. *oligofermentans* suspension cells after they were incubated in a buffer of pH 7.0 or pH 5.5 for 4 or 16 h. Table 1 shows that the CFU counts significantly dropped after incubating the cell suspension in a buffer of pH 5.5 for 16 h. No changes in CFU counts were observed under other incubation conditions. The *S*. *oligofermentans* suspension cells incubated in a buffer of pH 5.5 for 16 h demonstrated the highest potential of producing HP. As a result, the amount of HP produced per viable cell under this condition was about 15-fold higher than those incubated under other conditions.

**Table 1:** Viable cell counts and HP production of S. oligofermentans suspension cells after 4 or 16 h low pH challenge*.

Period	pH	Before incubation (logCFU)	After incubation (logCFU)	HP production (nM/viable cell)
4 h	7.0	8.0±0.1	7.9±0.1	4.3±0.3
	5.5	8.0±0.1	7.9±0.1	4.3±0.8
16 h	7.0	7.7±0.1	7.6±0.1	8.0±0.5
	5.5	7.6±0.1	6.5±0.1**	116.5±15.4**

* Data are presented as average ±Dsd.

** Statistical significance when compared to the corresponding values under neutral pH condition after 16 h incubation (*p* < 0.01).

## Discussion

In the oral cavity, the retention sites of the teeth, such as fissures and approximal sites, are prone to caries. The pH of dental biofilms at these sites drops rapidly upon intake of carbohydrates and can remain below pH 5.5 for a substantial period of time [18]. The prolonged low pH condition favours the aciduric bacterial species, such as cariogenic mutans streptococci and lactobacilli, which are able to survive and grow under this adverse condition [3,4]. Acid tolerance is considered the virulence trait for cariogenic bacteria to outcompete other microbes in dental biofilms. In this study, the competition between the cariogenic *S*. *mutans* and the commensal *S*. *oligofermentans* in dual-species biofilms was examined under either a constantly neutral pH or a pH-cycling condition (with a 16 h low pH period) in an active attachment biofilm model. Our data demonstrated that *S*. *oligofermentans* strongly inhibited the growth of *S*. *mutans* in dual-species biofilms and reduced lactic acid concentration at not only neutral pH condition, but also the pH-cycling condition. The inhibition of *S*. *oligofermentans* on the growth of *S*. *mutans* in dual-species biofilms has been reported in previous studies [8,10,11]. What is new in our finding is that the biofilms were formed through an active attachment process, while the previous studies formed the biofilms at the bottom of the wells. Since *S*. *mutans* is known to adhere better to a surface than other streptococci under sucrose condition, one could question if *S*. *oligofermentans* was still able to suppress *S*. *mutans* in the actively attached biofilms. The data from this study provided a positive answer to the question. Moreover, a recent study found that a low pH environment (pH<6.0) affected the ability of *S*. *oligofermentans* to inhibit the growth of *S*. *mutans* [12]. This result is contradictory to what we reported here. The discrepancy in these two studies might be explained by the experimental design. In the study of Liu *et al*. [12], the competition between two species was only tested on agar plates with various pH levels. Therefore, the growth of both bacterial species (*S*. *mutans* and *S*. *oligofermentans*) was affected by the constantly low pH, which made the competition between these two species impossible to observe. In our study, the oscillation of pH between 7.0 and 5.5 allowed the growth of both bacterial species. This design mimics the clinical condition better and makes it possible to study the competition between both species. Overall, our finding implied that *S*. *oligofermentans* might be a compelling probiotic candidate to compete against cariogenic bacterial species at sites prone to caries.

*S*. *mutans* is known to be acid tolerant. Previously, a panel of oral streptococci was tested for their acid tolerance [19–21]. All *S*. *mutans* strains were more aciduric than tested non-mutans streptococci, like *S*. *sanguinis*. Although substantial evidence has indicated that *S*. *sanguinis* and *S*. *gordonii* were capable of inhibiting the growth of *S*. *mutans*, these results were mostly achieved at a neutral pH environment. When a multi-species community was incubated under prolonged low pH condition, *S*. *mutans* would be favoured while *S*. *sanguinis* and *S*. *gordonii* would be suppressed [14,22]. *S*. *gordonii* was once tested as a probiotic for its inhibition on the growth of *S*. *mutans* in a rat caries model [23]. But *S*. *mutans* was found to overgrow *S*. *gordonii*, irrespective of the availability of sucrose and inoculation sequences. Therefore, *S*. *sanguinis* and *S*. *gordonii* may not be a good probiotic candidate to inhibit *S*. *mutans* at caries susceptible sites. The data presented here suggested that unlike *S*. *sanguinis* and *S*. *gordonii*, *S*. *oligofermentans* could inhibit *S*. *mutans* even after co-incubation at low pH for 16 h. This trait was indicated by the relatively high CFU counts of single-species *S*. *oligofermentans* biofilms under pH-cycling condition. It seems that *S*. *oligofermentans* is as acid resistant as *S*. *mutans*, since a similar level of CFU reduction was observed for both bacterial species when comparing pH-cycling condition with neutral pH condition.

Another intriguing finding of this study was the enhanced HP production of *S*. *oligofermentans* biofilms after growth under pH-cycling conditions. This was unexpected since the number of viable *S*. *oligofermentans* cells grown under pH-cycling conditions was much lower than of those grown under neutral pH conditions. There are a few possible explanations. The first one is that the presence of *S*. *mutans* in dual-species biofilms might trigger the HP production under pH-cycling conditions. But it could not explain why the HP production in single-species *S*. *oligofermentans* biofilms was as high as dual-species biofilms. The second explanation is that the high HP production was caused by the high lactate concentration, since *S*. *oligofermentans* is able to convert lactate into HP [9]. This assumption could explain the low lactate concentration observed in *S*. *oligofermentans* biofilms. But it could not explain why lactate concentration in *S*. *oligofermentans* biofilms was similar under both pH conditions, while HP production was different. The third explanation is related to the plating method used in this study. Since this method cannot detect those viable but not culturable bacterial cells, the unculturable *S*. *oligofermentans* cells in the biofilms might be the reason for the high HP production. We have carried out the metabolism (resazurin) assay to examine the metabolic activity of the tested biofilms, besides plating. This assay is able to detect viable bacterial cells without the requirement of further culturing. The results obtained from the assay were consistent with the results obtained from the plating method (data not shown). The fourth explanation is that the prolonged low pH incubation triggered the stress response of *S*. *oligofermentans* cells and resulted in enhanced HP production after the bacterial cells were incubated at neutral pH again. To explore this assumption, we carried out an additional experiment on *S*. *oligofermentans* suspension cells. Our data indicated 16 h, but not 4 h, mild acid pre-challenge could indeed increase the HP production per *S*. *oligofermentans* cell, even though the overall HP increase was not as pronounced as in biofilms.

In this study, we successfully established a high-throughput pH-cycling biofilm model. In order to establish a controllable pH-cycling condition, different buffers (phosphate or MES buffer) were added to the growth media. As a result, the pH of the biofilms could be maintained as designed. Since dental biofilms in the oral cavity constantly experience pH fluctuations, the established model can mimic this fluctuation condition and help to understand its influence on the bacterial interaction in dental biofilms. On the other hand, we also realized that the presented biofilm model, though well controlled, is too simple to fully mimic the complex oral conditions. Several important factors, such as dental hard tissues as substrata, salivary buffering, salivary flow or a multi-species community, should be taken into account in the model in the future studies. Eventually, the feasibility of *S*. *oligofermentans* as a probiotic under cariogenic condition should be evaluated in a more realistic model, such as an animal model.

In summary, the data presented here indicated that the non-mutans streptococci *S*. *oligofermentans* not only have the reported properties, including low acidogenic and being an HP producer, but also is aciduric and able to produce a substantial amount of HP after substantial acid challenge in the biofilms. These traits make *S*. *oligofermentans* the most competent candidate to compete against cariogenic bacteria *S*. *mutans in vivo*.

## Supporting Information

S1 Fig Colonies of *S*. *mutans* on BHI agar. The colony of *S*. *mutans* is white and with a rough surface.
(TIF)

S2 Fig Colonies of *S*. *oligofermentans* on BHI agar. The colony of *S*. *oligofermentans* is yellowish and flat.
(TIF)
