# Supplementary figures and images for: Streptococcus oligofermentans Inhibits Streptococcus mutans in Biofilms at Both Neutral pH and Cariogenic Conditions
Source: PLoS One. 2015 Jun 26;10(6):e0130962. doi: 10.1371/journal.pone.0130962 (PMC4483167; doi:10.1371/journal.pone.0130962)

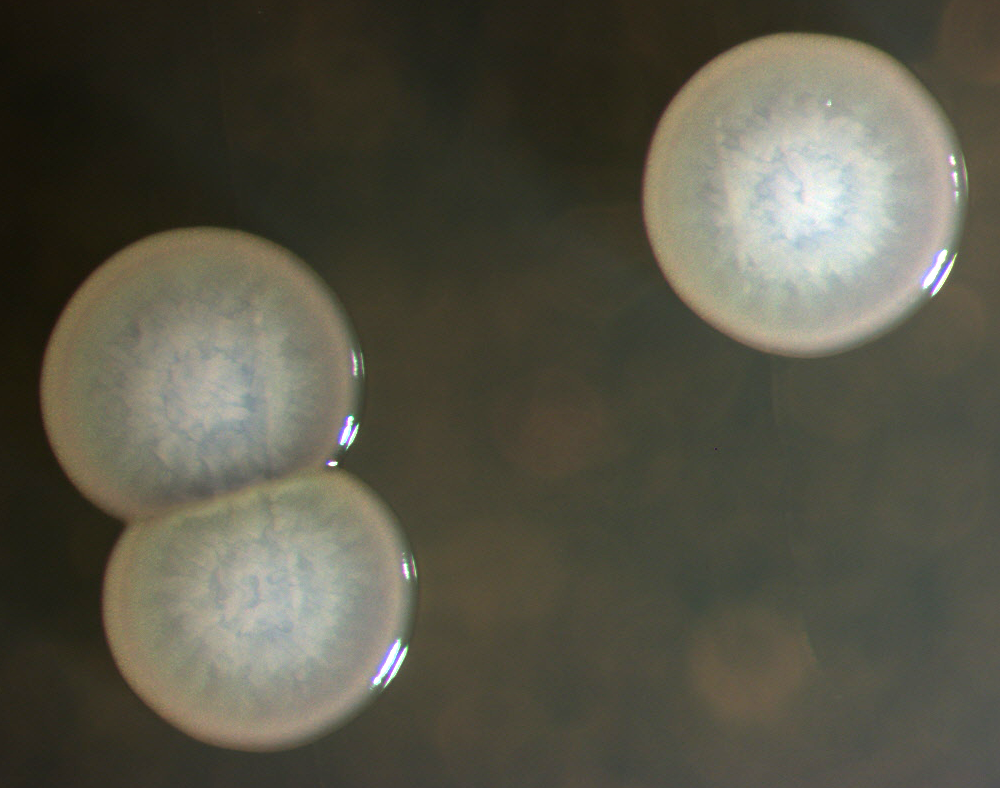

Supplement: S1 Fig — The colony of S. mutans is white and with a rough surface. (TIF) [file pone.0130962.s001.tif]

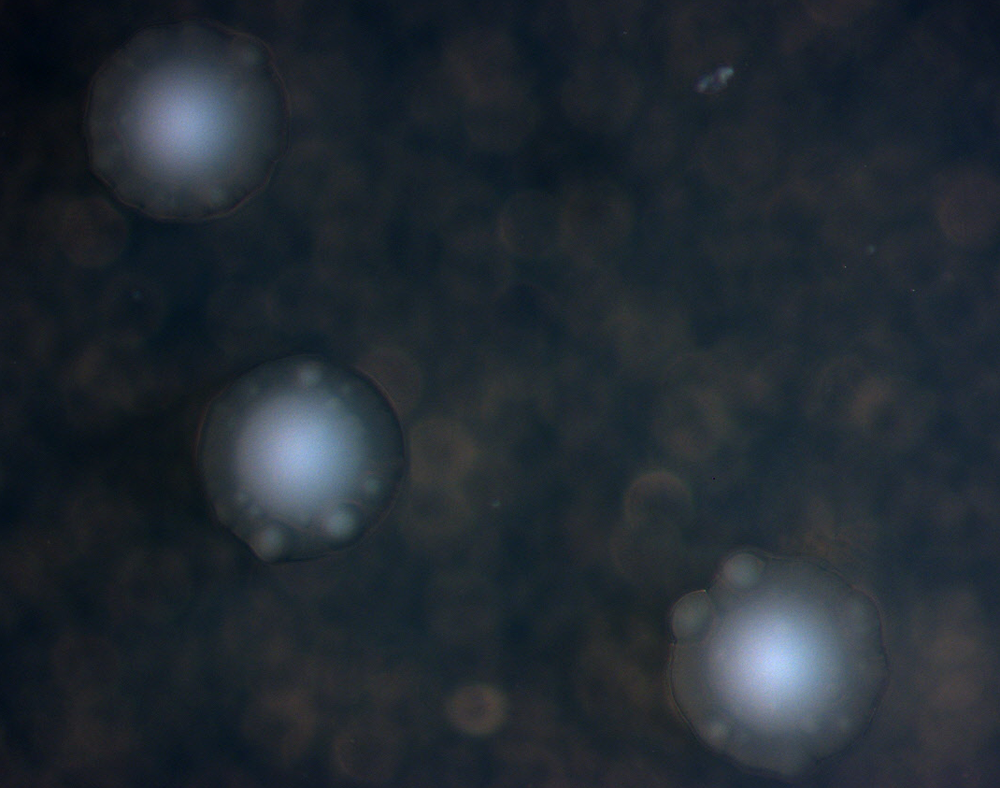

Supplement: S2 Fig — The colony of S. oligofermentans is yellowish and flat. (TIF) [file pone.0130962.s002.tif]
